# Spontaneous Splenic Rupture in Severe Acute Pancreatitis: A Rare Life-Threatening Complication and Its Successful Management

**DOI:** 10.7759/cureus.80354

**Published:** 2025-03-10

**Authors:** Mena Louis, Bolaji Ayinde, Brian Gibson

**Affiliations:** 1 General Surgery, Northeast Georgia Medical Center Gainesville, Gainesville, USA; 2 Internal Medicine, Northeast Georgia Medical Center Gainesville, Gainesville, USA; 3 Trauma and Acute Care Surgery, Northeast Georgia Medical Center Gainesville, Gainesville, USA

**Keywords:** acute pancreatitis, pancreatic pseudocysts, splenic artery embolization, splenic complications, spontaneous splenic rupture

## Abstract

The severity of acute pancreatitis ranges from mild discomfort to severe illness with significant complications. While most cases resolve with supportive care, severe acute pancreatitis may lead to rare but serious issues such as spontaneous splenic rupture. A 46-year-old female with a history of alcohol use, hypertension, depression, and anxiety presented with persistent abdominal pain, nausea, and vomiting. Initial imaging revealed acute pancreatitis with peripancreatic fluid collections. Despite conservative management, her symptoms persisted. She experienced sudden worsening of abdominal pain and a significant drop in hemoglobin levels. Imaging confirmed a spontaneous splenic rupture with a large subcapsular hematoma and hemoperitoneum. She underwent splenic artery embolization to control the bleeding and received blood transfusions for anemia. Her condition improved with supportive care, and she was discharged with plans for outpatient follow-up.

Spontaneous splenic rupture is a rare complication of acute pancreatitis resulting from the close anatomical relationship between the pancreas and spleen. Mechanisms behind it include direct enzymatic damage, pseudocyst extension, vascular injury, and increased pressure from splenic vein thrombosis. Early recognition is crucial for timely intervention. Clinicians should consider splenic complications when patients with pancreatitis exhibit sudden clinical deterioration or unexplained anemia. Prompt imaging and appropriate management can improve outcomes. Understanding the potential complications of severe pancreatitis is essential for effective patient care.

## Introduction

Acute pancreatitis is an inflammatory disorder of the pancreas characterized by the sudden onset of abdominal pain and elevated levels of pancreatic enzymes in the blood (serum amylase: 999 U/L, normal range: 30-110 U/L; serum lipase: 535 U/L, normal range: 10-140 U/L) [1]. It is a leading cause of gastrointestinal hospital admissions worldwide, presenting a wide clinical spectrum from mild discomfort to severe, life-threatening conditions [2]. While most patients experience a self-limiting course that responds well to supportive care, a significant minority develop severe acute pancreatitis associated with high morbidity and mortality [3]. Severe acute pancreatitis can lead to a range of complications that profoundly affect patient outcomes [4]. Local complications include pancreatic necrosis, formation of pseudocysts, and vascular issues such as hemorrhage and thrombosis [5]. Systemic complications may involve multiple organ systems, resulting in organ failure and systemic inflammatory response syndrome [6]. Among the rare but critical local complications is spontaneous splenic rupture, which arises due to the anatomical proximity of the pancreas to the spleen [7].

## Case presentation

A 46-year-old female with a history of hypertension, depression, anxiety, and prior alcohol use presented to the emergency department with severe abdominal pain, nausea, and vomiting. Her symptoms had begun approximately one month earlier when she had been diagnosed with acute pancreatitis at another hospital, where imaging had revealed pancreatitis and two pancreatic cysts. She had been hospitalized for nine days, managed conservatively, and discharged 10 days before the current presentation. Despite being advised to follow up with a gastroenterologist, she had been unable to secure an appointment. Her abdominal discomfort, nausea, and vomiting persisted and worsened on the day of admission.

On examination, she reported diffuse abdominal pain rated 10/10. She denied chest pain, shortness of breath, and changes in bowel or urinary habits, and confirmed abstinence from alcohol since the onset of her illness. Vital signs showed a blood pressure of 164/108 mmHg, a heart rate of 100 beats per minute, and she was afebrile. Physical examination revealed a distended abdomen with diffuse tenderness.

**Figure 1:**
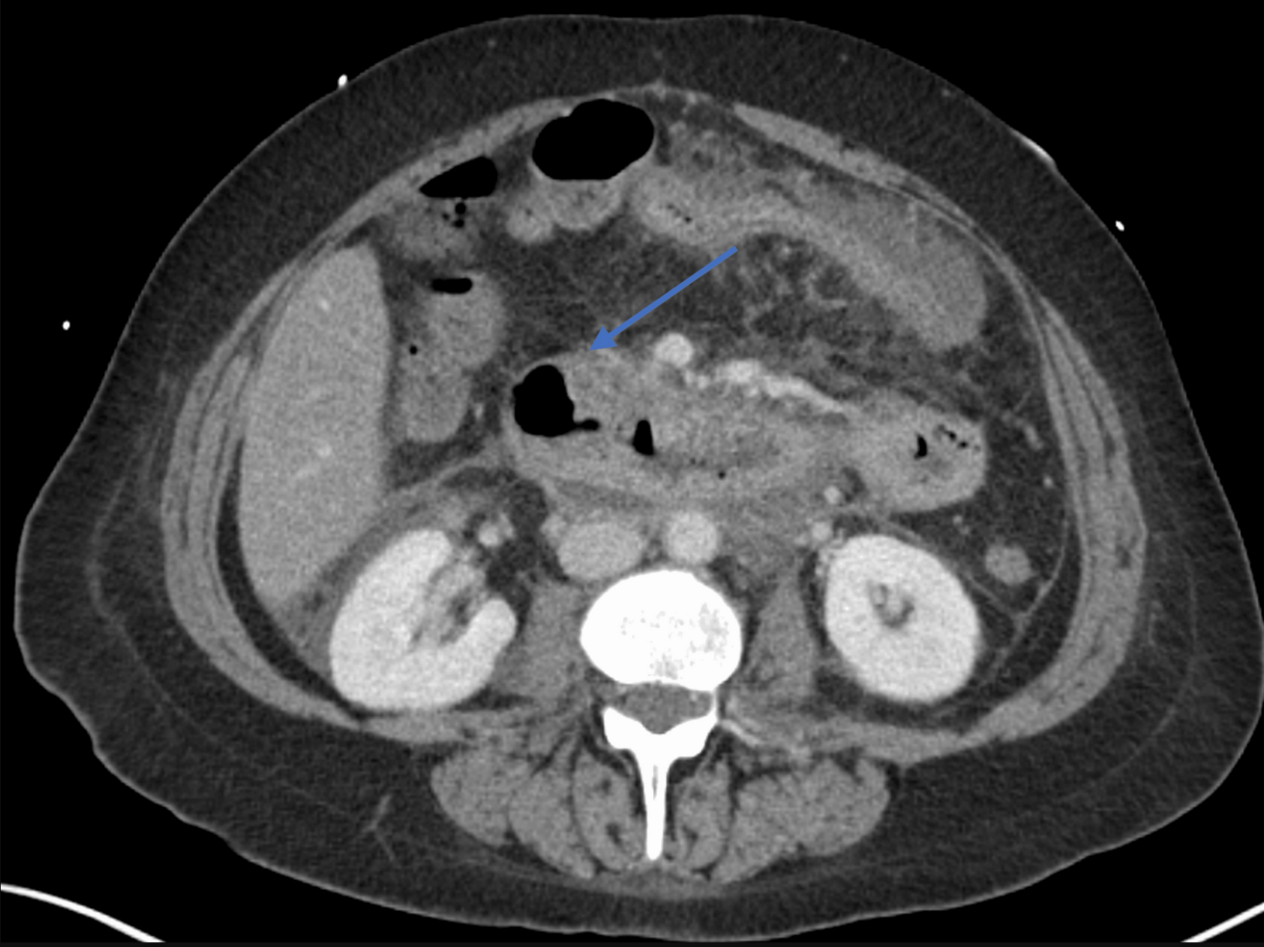
CT scan of the abdomen with IV contrast (axial view) demonstrating pancreatitis and multiple peripancreatic fluid collections consistent with severe acute pancreatitis CT: computed tomography

Initial laboratory tests indicated anemia with a hemoglobin level of 10.5 g/dL, which decreased to 8.8 g/dL over the next few days. Electrolyte imbalances included hyponatremia and hypokalemia (Table 1). A CT scan of the abdomen and pelvis showed findings consistent with acute pancreatitis and multiple peripancreatic fluid collections (Figure 1), including an 8.0 × 5.0 cm lesion anterior to the left lobe of the liver suggestive of a pseudocyst or abscess.

**Table 1:** Summary of key laboratory findings ALT: alanine aminotransferase; AST: aspartate aminotransferase

Parameter	Initial value	Lowest value	Normal range
Hemoglobin (g/dL)	10.5	8.8	12.0–16.0
Hematocrit (%)	31.2	26.3	36.0–46.0
White blood cell count (×10⁹/L)	8.8	7.6	4.0–11.0
Sodium (mEq/L)	136	130	135–145
Potassium (mEq/L)	3.1	3.2	3.5–5.0
Albumin (g/dL)	2.1	1.9	3.5–5.0
Total bilirubin (mg/dL)	0.44	0.36	0.1–1.2
AST (U/L)	17	20	10–40
ALT (U/L)	<9	<9	7–56
Creatinine (mg/dL)	0.49	0.41	0.6–1.2
Glucose (mg/dL)	115	89	70–140

The patient was admitted for the management of acute pancreatitis and started on intravenous fluids, pain control with analgesics, and pancreatic enzyme supplementation. However, she continued to experience severe abdominal pain and had episodes of fever reaching 102 °F. A hepatobiliary surgery consultation recommended discontinuing prophylactic antibiotics, as there was no evidence of infected pancreatic necrosis or another confirmed bacterial infection. Despite the presence of systemic inflammatory response syndrome (SIRS) - which is characterized by at least two of the following criteria: fever or hypothermia (>38 °C or <36 °C), tachycardia (>90 bpm), tachypnea (>20 breaths per minute), or leukocytosis/leukopenia (WBC >12,000 or <4,000 cells/mm³) - current guidelines do not support routine antibiotic use in acute pancreatitis unless an infection is identified. Prokinetic agents were initiated for delayed gastric emptying, and the patient was advanced to a regular low-fat diet as tolerated.

**Figure 2:**
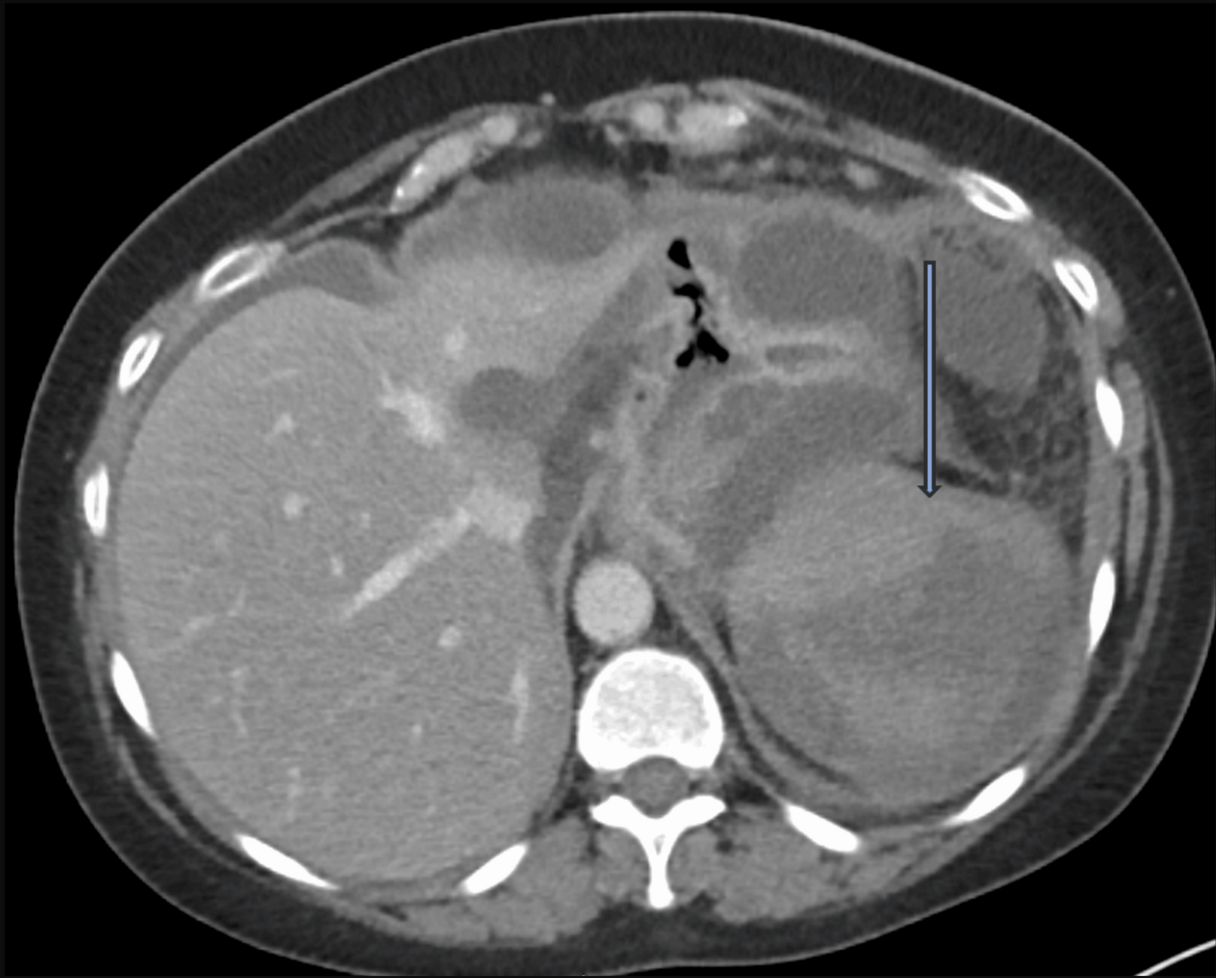
CT scan of the abdomen demonstrating spontaneous splenic rupture with a large subcapsular hematoma, perisplenic hematoma, and hemoperitoneum The blue arrow points at the large subcapsular hematoma CT: computed tomography

Nine days into her hospital stay, she experienced a sudden worsening of abdominal pain, accompanied by nausea and vomiting. Laboratory results showed a significant drop in hemoglobin to 5.9 g/dL and an elevated white blood cell count of 24.0 × 10⁹/L, raising concern for hemorrhagic complications. A repeat CT scan revealed splenic rupture secondary to pancreatitis, with a large subcapsular hematoma, perisplenic hematoma, and hemoperitoneum, along with worsening peripancreatic fluid collections (Figure 2).

The patient underwent urgent splenic artery embolization, which successfully controlled the bleeding. Following the procedure, her hemoglobin levels stabilized after blood transfusions, and her abdominal pain gradually improved. She was able to tolerate a low-fat diet without recurrent nausea or vomiting. Given her stable condition, she was discharged with plans for outpatient follow-up to monitor the pancreatic fluid collections and assess for any long-term complications related to disconnected pancreatic duct syndrome.

## Discussion

Acute pancreatitis is an inflammatory condition of the pancreas with a spectrum ranging from mild discomfort to severe illness involving multiple organ systems [8]. While most cases are self-limiting, severe acute pancreatitis can lead to significant local and systemic complications that increase morbidity and mortality [9]. In our patient, the persistent abdominal pain, nausea, and vomiting were consistent with acute pancreatitis. Her history of alcohol use is a well-established risk factor, contributing to a significant number of pancreatitis cases worldwide.

The diagnosis of acute pancreatitis is established when at least two of the following three criteria are met: (1) abdominal pain characteristic of pancreatitis, (2) serum amylase and/or lipase levels elevated to at least three times the upper limit of normal, and (3) imaging findings consistent with pancreatitis. Our patient met all three criteria, with severe epigastric pain, a serum amylase of 999 U/L (normal: 30-110 U/L) and lipase of 535 U/L (normal: 10-140 U/L), and CT evidence of pancreatic inflammation and peripancreatic fluid collections. Despite initial conservative management and abstinence from alcohol, she developed multiple peripancreatic fluid collections, indicating a progression to a more severe form of the disease.

Spontaneous splenic rupture is a rare but serious complication of acute pancreatitis. The close anatomical relationship between the pancreas, particularly the tail, and the spleen allows inflammatory processes to affect splenic tissue [10]. Mechanisms for splenic involvement include direct erosion by pancreatic enzymes, extension of pseudocysts into the spleen, vascular damage leading to infarction, and increased pressure from splenic vein thrombosis or localized portal hypertension [11]. The sudden worsening of her abdominal pain accompanied by a significant drop in hemoglobin levels raised suspicion for intra-abdominal hemorrhage. Imaging confirmed a large subcapsular and perisplenic hematoma consistent with splenic rupture, as well as worsening peripancreatic fluid collections. Prompt intervention was necessary to control bleeding and prevent hemodynamic instability. Splenic artery embolization was effectively utilized, preserving splenic function and avoiding surgical removal.

Peripancreatic fluid collections and pseudocysts are common complications of pancreatitis resulting from leakage of pancreatic enzymes and inflammatory exudate into surrounding tissues. While many pseudocysts resolve spontaneously, larger or symptomatic collections may require interventions [12]. In this case, the fluid collections were managed conservatively with close monitoring, as there were no signs of infection or compression of adjacent structures.

Severe acute pancreatitis can lead to a range of complications that require timely recognition and management. While pancreatic necrosis and peripancreatic fluid collections are more commonly encountered, vascular complications such as splenic rupture are less frequent but can be life-threatening. In this case, the patient's sudden drop in hemoglobin and worsening abdominal pain raised concern for hemorrhagic complications, prompting urgent imaging. Contrast-enhanced CT played a crucial role in identifying the subcapsular and perisplenic hematoma, guiding the decision for splenic artery embolization. Clinicians should consider splenic complications in patients with pancreatitis who present with unexplained anemia, hypotension, or left upper quadrant pain, as early diagnosis and intervention can improve outcomes [13].

Various studies have documented the range of complications associated with severe acute pancreatitis. Common issues include pancreatic necrosis, infected pseudocysts, and organ failure, all contributing to increased morbidity and mortality [14]. Vascular complications, though less common, pose significant risks and require timely intervention. Early recognition of these complications is crucial, as they can be life-threatening if not promptly diagnosed and managed. Clinicians should maintain a high index of suspicion and utilize appropriate imaging modalities to detect splenic involvement in patients with acute pancreatitis [15,16]. 

Peripancreatic fluid collections are common in acute pancreatitis and are classified based on the Revised Atlanta Classification (Table 2) [17]. These collections can evolve into pseudocysts or walled-off necrosis, depending on the presence of necrotic material. Management strategies depend on the size, location, and symptoms caused by the fluid collections. Conservative management is often sufficient, but intervention may be necessary if complications develop.

**Table 2:** Revised Atlanta classification of acute pancreatitis fluid collections

Type of collection	Time of onset, weeks	Contents	Wall formation	Characteristics
Acute peripancreatic fluid collection (APFC)	<4	Fluid only	No wall	Typically sterile, seen in interstitial edematous pancreatitis
Pancreatic pseudocyst	>4	Fluid only	Well-defined wall	Develops from APFC if persistent
Acute Necrotic collection (ANC)	<4	Necrotic material ± fluid	No wall	Seen in necrotizing pancreatitis, may involve pancreas or peripancreatic tissues
Walled-off necrosis (WON)	>4	Necrotic material ± fluid	Well-defined wall	Develops from ANC if persistent, may require drainage if symptomatic or infected

Vascular complications in pancreatitis include pseudoaneurysm formation, hemorrhage, and thrombosis of adjacent vessels [18]. Arterial pseudoaneurysms can result from enzymatic degradation of the vessel wall, leading to potential rupture and hemorrhage. Endovascular techniques such as embolization have become the preferred treatment due to their minimally invasive nature and high success rates. 

Managing severe acute pancreatitis and its complications requires a multidisciplinary approach [19]. Early aggressive fluid resuscitation, pain control, and nutritional support are fundamental. The use of prophylactic antibiotics is debated but indicated in confirmed infections. Interventional radiology plays a crucial role in managing vascular complications, providing less invasive options with reduced morbidity compared to surgical interventions.

## Conclusions

Severe acute pancreatitis can lead to rare but serious complications that require prompt recognition and management. Clinicians should remain vigilant for signs of vascular involvement, such as spontaneous splenic rupture, especially when patients exhibit sudden clinical deterioration or unexplained anemia. Early diagnosis through imaging and timely intervention can significantly improve patient outcomes. A multidisciplinary approach involving gastroenterology, surgery, radiology, and critical care teams is essential for effective management. Understanding the potential complications and their underlying mechanisms is crucial for optimizing care and reducing morbidity and mortality associated with severe pancreatitis.
